# Delay in reviewing test results prolongs hospital length of stay: a retrospective cohort study

**DOI:** 10.1186/s12913-018-3181-z

**Published:** 2018-05-16

**Authors:** Mei-Sing Ong, Farah Magrabi, Enrico Coiera

**Affiliations:** 1 Department of Population Medicine, Harvard Medical School and Harvard Pilgrim Health Care Institute, 401 Park Drive, Suite 401E, Boston, MA 02115 USA; 2 Australian Institute of Health Innovation, Macquarie University, Sydney, Australia

**Keywords:** Length of stay, Test results, Delay

## Abstract

**Background:**

Failure in the timely follow-up of test results has been widely documented, contributing to delayed medical care. Yet, the impact of delay in reviewing test results on hospital length of stay (LOS) has not been studied. We examine the relationship between laboratory tests review time and hospital LOS.

**Methods:**

A retrospective cohort study of inpatients admitted to a metropolitan teaching hospital in Sydney, Australia, between 2011 and 2012 (*n* = 5804). Generalized linear models were developed to examine the relationship between hospital LOS and cumulative clinician read time (CRT), defined as the time taken by clinicians to review laboratory test results performed during an inpatient stay after they were reported in the computerized test reporting system. The models were adjusted for patients’ age, sex, and disease severity (measured by the Charlson Comorbidity index), the number of test panels performed, the number of unreviewed tests pre-discharge, and the cumulative laboratory turnaround time (LTAT) of tests performed during an inpatient stay.

**Results:**

Cumulative CRT is significantly associated with prolonged LOS, with each day of delay in reviewing test results increasing the likelihood of prolonged LOS by 13.2% (*p* < 0.0001). Restricting the analysis to tests with abnormal results strengthened the relationship between cumulative CRT and prolonged LOS, with each day of delay in reviewing test results increasing the likelihood of delayed discharge by 33.6% (*p* < 0.0001). Increasing age, disease severity and total number of tests were also significantly associated with prolonged LOS. Increasing number of unreviewed tests was negatively associated with prolonged LOS.

**Conclusions:**

Reducing unnecessary hospital LOS has become a critical health policy goal as healthcare costs escalate. Preventing delay in reviewing test results represents an important opportunity to address potentially avoidable hospital stays and unnecessary resource utilization.

**Electronic supplementary material:**

The online version of this article (10.1186/s12913-018-3181-z) contains supplementary material, which is available to authorized users.

## Background

Inpatient hospital services constitute almost one-third of all health care expenditures in the United States [1]. Substantial research and policy efforts have been directed at reducing unnecessary hospital length of stay (LOS), a key indicator for inpatient resource use. Research into the determinants of hospital length of stay (LOS) has identified a range of patient characteristics and clinical predictors contributing to prolonged LOS [2]. These include age, sex, disease severity, presenting conditions and complications [3–10]. While useful in guiding resource planning, most patient and disease-specific variables are not amenable to interventions that would reduce unnecessary hospital stays.

More often than not, hospital LOS is also driven by institutional factors unrelated to individual patients’ conditions, such as clinical workflow and hospital resources. Patients may experience prolonged hospitalizations due to delays in accessing care [11]. Identifying these modifiable factors can provide important opportunities for interventions.

Here, we conduct the first study to explore the relationship between laboratory tests review time and LOS. Because clinical laboratory testing is an integral part of healthcare delivery, driving a large proportion of critical medical decisions, we hypothesize that delay in reviewing test results prolongs hospital LOS.

## Methods

### Settings and participants

This study was conducted at a 370-bed metropolitan teaching hospital. A hospital-wide computerized system was in place for reporting all diagnostic tests. The system captured detailed information on the time and date when laboratory tests were requested, when the results were available for review, and when they were viewed by the providers. Participants included all inpatients admitted to the hospital between February 2011 and February 2012. Clinical pathology tests performed were extracted from the computerized test-reporting system; these included clinical chemistry, clinical pharmacology, hematology, immunology and microbiology. Tests relating to arterial blood gases were excluded, since the results for these tests were communicated directly to the ordering physician. We further extracted from the hospital admission database the admission details of the patients, including date of admission and discharge, principal diagnosis (expressed as free text), and the department where inpatient care was provided. This study was approved by the Human Research Ethics Committee at the University of New South Wales (Sydney, Australia), with patient consent waived.

### Study outcomes

**Fig. 1:**
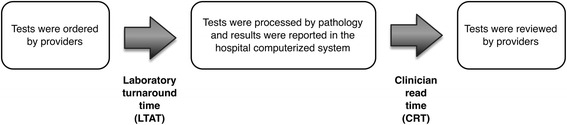
Laboratory tests processing and review workflow

The primary outcome of interest was the relationship between hospital LOS and clinician read time (CRT), defined as the time taken by a provider to access laboratory test results after they were available for review in the test reporting system (Fig. 1). The provider who reviewed the test results may be the ordering provider or any members of the healthcare team. Since patients often underwent multiple tests, we calculated the cumulative CRT for all tests performed during an inpatient stay.

Timeliness in reviewing test results is dependent on timely processing of laboratory tests. The impact of laboratory turnaround time (LTAT), defined as the time between ordering of test and when the test results are available for review, has been reported to prolong LOS in high-volume patient care settings such as the emergency department. We therefore assessed the modifying effect of LTAT on the relationship between CRT and hospital LOS, and compare the effects of CRT and LTAT on hospital LOS. We hypothesize that cumulative CRT is independently associated with prolonged hospital LOS.

### Statistical analysis

Descriptive statistics were used to quantify the prevalence of baseline characteristics, including patient demographics, inpatient medical specialties, the time of day tests were ordered, processed and reviewed. The impact of CRT on LOS was evaluated using generalized linear models. We used the gamma family of distributions to account for skewness in the distribution of LOS, with logarithm as the link function. We included in the model patients’ age, sex, and disease severity (measured by the Charlson Comorbidity index), the total number of test panels performed during the inpatient stay, and the cumulative LTAT. We further controlled for the number of tests that were not reviewed during the inpatient stay, since tests that were not followed up were unlikely to prolong hospital LOS. An additional sensitivity analysis was performed that excluded patients with tests that were not followed up during an inpatient stay. To compare the impact of delay in reviewing abnormal test results and normal test results, we conducted a sensitivity analysis that considered only tests with abnormal findings, and a separate analysis that included only normal test results. With the exception of sex, all variables were modeled as continuous variables. The resulting estimated coefficients are presented as back-transformed (exponentiated), which are interpretable as the multiplicative effects of predictor variables on the expected LOS.

To address potential reverse causation in the association between CRT and LOS, whereby prolonged LOS causes delay in CRT, we assessed variability in the follow-up behavior of providers throughout inpatient stays, by quantifying the average CRT for tests ordered on each day of hospital admission, from day one to day 14 of admission. We observed that tests ordered on the first 2 days of admission had an average CRT of 26 and 21 h, respectively (Additional file 1: Figure S1). The average CRT for tests ordered on subsequent days (i.e. day three to 14 of admission) was about 12 h and did not vary with prolonged LOS. To account for this pattern, we conducted an additional sensitivity analysis where CRT was measured based only on tests ordered in the first 48 h of admission, and a further analysis that quantified CRT based on tests ordered after the first 48 h of admission among patients who were hospitalized for two or more days.

Analyses were conducted using R statistical software version 3.4.3. All tests of statistical significance were two-tailed and used an α level of *p* < 0.05.

### Subgroup analyses

Clinical workflow and resources may differ across departments within the hospital, which may alter the relationship between CRT and hospital LOS. To assess if the effect of CRT on hospital LOS was consistently observed across different medical departments, we performed subgroup analyses for four of the departments with the largest number of inpatient admissions during the study period: geriatrics, cardiology, general surgery, and psychiatry. Separate models were developed for each department to assess the relationship between cumulative CRT and LOS. To account for potential confounding associated with patient heterogeneity, we further performed additional subgroup analyses that considered inpatients with the same principal admitting diagnosis. Two subgroups were selected a priori: patients with renal failure and patients who underwent elective knee or hip replacement surgery; the former represents a group of critically ill patients requiring urgent care, and the latter represents a more routine and non-urgent patient population. We hypothesize that the impact of CRT on LOS was greater among the renal failure population, compared with patients who were admitted for an elective knee or hip replacement surgery.

## Results

**Fig. 2:**
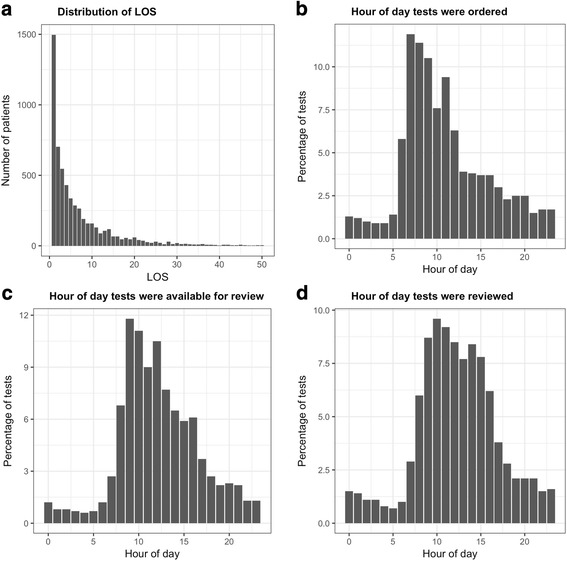
Distribution of hospital length of stay (LOS) and patterns of test ordering, processing and review

A total of 5804 inpatient admissions satisfied the inclusion criteria (Table 1). The mean and median LOS were 7.5 and 4 days, respectively. The distribution of LOS is illustrated in Fig. 2a. On average, 7.2 test panels were performed during an inpatient stay, with a median of 4 test panels per inpatient stay. An inpatient experienced a mean cumulative CRT of 1.6 days, and a cumulative LTAT of 2.5 days. The average CRT and LTAT for individual tests was 12.7 h and 16.1 h, respectively; the median CRT and LTAT for individual tests was an hour.

**Table 1:** Study participants (*n* = 5804)

Attributes	N (%)
Age	
< = 30	140 (2.4)
31 to 64	2530 (43.6)
> = 65	3134 (54.0)
Sex (female)	2481 (42.7)
Charlson comorbidity index	
0	3508 (60.4)
1	897 (15.5)
2	649 (11.2)
3	318 (5.5)
> = 4	432 (7.4)
Patients with one or more unreviewed tests, n (%)	3129 (53.9)
Department, n (%)	
Geriatrics	983 (16.9)
Cardiology	765 (13.2)
General surgery	554 (9.5)
Psychiatry	396 (6.8)
Respiratory medicine	388 (6.7)
Orthopedic surgery	354 (6.1)
Neurology	237 (4.1)
Gastroenterology	217 (3.7)
Vascular surgery	155 (2.7)
Neurosurgery	150 (2.6)
Hematology	147 (2.5)
Urology	144 (2.5)
Cardiothoracic surgery	108 (1.9)
Nephrology	102 (1.8)
Dermatology	75 (1.3)
Rehabilitation	68 (1.2)
Otolaryngology	62 (1.1)
Palliative care	57 (1.0)
Drug and alcohol	53 (0.9)
Immunology	50 (0.9)
Infectious diseases	49 (0.8)
Endocrinology	32 (0.6)
Oncology	29 (0.5)
Obstetrics and gynecology	23 (0.4)
Rheumatology	23 (0.4)
Maxillofacial surgery	6 (0.1)
Dental surgery	2 (0.03)
Radiation oncology	2 (0.03)
Ophthalmology	1 (0.02)
Unspecified	572 (9.9)

Cumulative CRT is significantly associated with prolonged LOS, with each day of delay in reviewing test results increasing the likelihood of prolonged hospital stay by 13.2% (*p* < 0.0001). Increasing age, disease severity and total number of tests were also significantly associated with prolonged LOS (Table 2a). Patient’s sex was not associated with LOS in univariate analysis, and was therefore excluded from the multivariate models. While cumulative LTAT was significantly associated with LOS in univariate analysis, its effect on LOS became insignificant in the multivariate generalized linear model. Increasing number of unreviewed tests was negatively associated with prolonged LOS.

**Table 2:** Generalized linear model for predicting hospital LOS

Covariate	Estimate (β) (95% CI)	*p*-value
(a) Primary analysis including all inpatients		
Age	1.003 (1.001–1.005)	< 0.0001
Charlson comorbidity index	1.020 (1.004–1.037)	0.017
Number of test panels	1.054 (1.048–1.060)	< 0.0001
Cumulative CRT (days)	1.132 (1.116–1.149)	< 0.0001
Cumulative LTAT (days)	0.996 (0.988–1.004)	0.165
Number of unreviewed tests	0.988 (0.985–0.992)	< 0.0001
(b) Sensitivity analysis including only patients without any unreviewed tests		
Age	1.001 (0.999–1.004)	0.267
Charlson comorbidity index	1.019 (0.989–1.051)	0.247
Number of test panels	1.060 (1.047–1.073)	< 0.0001
Cumulative CRT (days)	1.186 (1.154–1.221)	< 0.0001
Cumulative LTAT (days)	1.028 (1.004–1.054)	0.022
(c) Sensitivity analysis including only laboratory tests with abnormal results		
Age	1.001 (0.999–1.002)	0.381
Charlson comorbidity index	1.020 (1.004–1.037)	0.022
Number of test panels	1.073 (1.065–1.081)	< 0.0001
Cumulative CRT (days)	1.336 (1.294–1.380)	< 0.0001
Cumulative LTAT (days)	1.010 (0.993–1.029)	0.231
Number of unreviewed tests	0.971 (0.958–0.984)	< 0.0001
(d) Sensitivity analysis including only laboratory tests with normal results		
Age	1.003 (1.001–1.004)	0.002
Charlson comorbidity index	1.019 (1.003–1.036)	0.026
Number of test panels	1.063 (1.056–1.070)	< 0.0001
Cumulative CRT (days)	1.122 (1.105–1.139)	< 0.0001
Cumulative LTAT (days)	0.990 (0.982–0.999)	0.002
Number of unreviewed tests	0.984 (0.980–0.988)	< 0.0001
(e) Sensitivity analysis including only tests ordered in the first two days of admission		
Age	1.003 (1.001–1.005)	0.002
Charlson comorbidity index	1.018 (1.001–1.037)	0.057
Number of test panels	1.065 (1.058–1.072)	< 0.0001
Cumulative CRT (days)	1.030 (1.017–1.044)	< 0.0001
Cumulative LTAT (days)	1.017 (1.008–1.027)	< 0.0001
Number of unreviewed tests	0.987 (0.983–0.991)	< 0.0001
(f) Sensitivity analysis excluding tests ordered in the first two days of admission		
Age	1.001 (1.000–1.003)	0.159
Charlson comorbidity index	0.999 (0.984–1.013)	0.855
Number of test panels	1.025 (1.020–1.031)	< 0.0001
Cumulative CRT (days)	1.055 (1.046–1.064)	< 0.0001
Cumulative LTAT (days)	0.990 (0.983–0.997)	0.001
Number of unreviewed tests	0.989 (0.986–0.992)	< 0.0001

A total of 3129 (53.9%) patients had one or more test results that were not followed up during hospitalization. Excluding these patients from the analysis strengthened the impact of cumulative CRT on prolonged LOS, with each day of delay in reviewing test results increasing the likelihood of delayed discharge by 18.6% (*p* < 0.0001). In this subpopulation, increasing age and disease severity were not associated with prolonged LOS; increasing number of test panels and cumulative LTAT were associated with prolonged LOS (Table 2b).

Of the laboratory tests ordered, 30.4% had abnormal results. Including only tests with abnormal results in the analysis strengthened the relationship between cumulative CRT and prolonged LOS, with each day of delay in reviewing test results increasing the likelihood of delayed discharge by 33.6% (*p* < 0.0001), compared with 12.2% (*p* < 0.0001) when only tests with normal results were considered (Table 2c, d). In the two sensitivity analyses that quantified delay based on tests ordered within the first 48 h of admission and tests ordered after the first 48 h of admission, respectively, cumulative CRT remained a significant predictor of LOS (*p* < 0.0001) (Table 2e, f).

### Subgroup analyses

The effect of cumulative CRT on prolonged LOS was consistently observed across different departments (geriatrics, cardiology, general surgery and psychiatry) and patient subgroups with varying principal diagnoses (renal failure, knee or hip replacement) (Table 3). Increasing number of tests performed during inpatient stay was also consistently associated with prolonged LOS. The relationship between prolonged LOS and the remaining covariates were variably observed across departments and principal diagnoses.

**Table 3:** Subgroup analyses by department and principal diagnoses

Covariate	Estimate (β) (95% CI)	*p*-value
(a) Department: geriatric medicine		
Age	0.998 (0.991–1.005)	0.516
Charlson comorbidity index	0.992 (0.957–1.029)	0.661
Number of test panels	1.072 (1.060–1.085)	< 0.0001
Cumulative CRT (days)	1.108 (1.080–1.137)	< 0.0001
Cumulative LTAT (days)	0.995 (0.972–1.018)	0.652
Number of unreviewed tests	0.996 (0.990–1.003)	0.274
(b) Department: cardiology medicine		
Age	1.001 (0.998–1.004)	0.467
Charlson comorbidity index	1.049 (1.018–1.083)	0.002
Number of test panels	1.112 (1.099–1.126)	< 0.0001
Cumulative CRT (days)	1.049 (1.012–1.092)	0.001
Cumulative LTAT (days)	0.979 (0.962–0.998)	0.003
Number of unreviewed tests	0.999 (0.993–1.006)	0.829
(c) Department: general surgery		
Age	1.003 (0.999–1.006)	0.123
Charlson comorbidity index	1.019 (0.986–1.056)	0.283
Number of test panels	1.114 (1.097–1.132)	< 0.0001
Cumulative CRT (days)	1.034 (1.000–1.076)	0.026
Cumulative LTAT (days)	0.975 (0.944–1.008)	0.133
Number of unreviewed tests	0.992 (0.986–0.998)	0.012
(d) Department: psychiatry		
Age	1.011 (1.000–1.023)	0.054
Charlson comorbidity index	1.060 (0.951–1.212)	0.368
Number of test panels	1.168 (1.080–1.270)	< 0.0001
Cumulative CRT (days)	1.140 (1.077–1.213)	< 0.0001
Cumulative LTAT (days)	1.048 (0.995–1.112)	0.031
Number of unreviewed tests	0.959 (0.942–0.981)	0.0002
(e) Principal diagnosis: renal failure		
Age	1.007 (0.995–1.019)	0.289
Charlson comorbidity index	0.921 (0.841–1.013)	0.087
Number of test panels	1.037 (0.995–1.080)	0.090
Cumulative CRT (days)	1.182 (1.092–1.289)	0.0002
Cumulative LTAT (days)	1.006 (0.966–1.050)	0.776
Number of unreviewed tests	0.994 (0.977–1.013)	0.509
(f) Principal diagnosis: knee or hip replacement surgery		
Age	1.004 (0.994–1.014)	0.404
Charlson comorbidity index	1.025 (0.848–1.265)	0.815
Number of test panels	1.054 (1.016–1.095)	0.007
Cumulative CRT (days)	1.177 (1.071–1.299)	0.003
Cumulative LTAT (days)	1.004 (0.905–1.116)	0.941
Number of unreviewed tests	0.992 (0.978–1.008)	0.335

### Clinical workflow

The majority of inpatient laboratory tests were ordered between 6 am and 12 pm (Fig. 2b), and most test results were available for review between 8 am and 2 pm (Fig. 2c). Review of test results followed the same trend, peaking between 9 am and 3 pm (Fig. 2d).

**Fig. 3:**
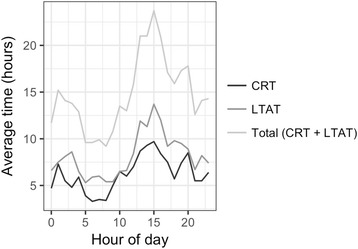
The relationship between hour of day tests were ordered, and the laboratory turnaround time (LTAT), clinician read time (CRT), and the total time (LTAT + CRT)

Figure 3 depicts the relationship between the time of day a laboratory test was placed, and the average CRT and LTAT, considering only tests that were reviewed during the inpatient stay. Both CRT and LTAT appeared to peak at around 2 pm, where the average total time taken to process and review a test was 23.7 h after a test was ordered. Comparing the CRT and LTAT of laboratory tests ordered before and after 2 pm, those that were placed after 2 pm experienced a much greater total delay (18.0 h vs 12.4 h; *p* < 0.0001), with both a greater CRT (7.5 vs 5.2 h; *p* < 0.0001) and LTAT (10.1 vs 6.8 h; *p* < 0.0001).

## Discussion

We established for the first time that delay in reviewing laboratory test results is significantly associated with prolonged hospital LOS. Delay in reviewing test results may prevent timely diagnosis and treatment, resulting in suboptimal outcomes that may further contribute to prolonged LOS. In our study, only one-third of test results had abnormal findings. Thus, in most cases, delay in test results follow-up was unlikely to affect health outcomes. However, our analysis shows that delay in reviewing normal test results is also a significant predictor of prolonged hospitalization. Patients may remain hospitalized while awaiting their test results to be reviewed before a discharge decision can be made. Reducing test review delay therefore represents an important opportunity to address potentially avoidable hospital stays and unnecessary resource utilization.

Turnaround time for laboratory tests did not appear to affect hospital LOS in the primary analysis that included all inpatients. A probable explanation is that a large proportion of laboratory tests were not actually reviewed prior to patient discharge [12] – delay in processing these tests were therefore unlikely to influence LOS. Indeed, the number of unreviewed tests was found to be negatively associated with LOS. In the secondary analysis that considered only patients who had their test results reviewed during their inpatient stay, a significant relationship between increasing cumulative LTAT and prolonged LOS was observed. However, the strength of the association was notably weaker than the association between delay in reviewing test results and LOS. The effects of LTAT on LOS have been extensively studied, primarily in the emergency care setting [13]. While a number of studies demonstrated a significant relationship between LTAT and LOS, others have failed to show the benefit of reducing LTAT on LOS [14, 15]. Substantial efforts to improve the efficiency of pathology workflow has significantly reduced the delay associated with laboratory turnaround time. However, delay in reviewing test results has received much less attention. Our analysis suggests that unless test results are reviewed in a timely manner, any benefits gained from reducing LTAT will be greatly diminished.

The time of day laboratory tests were ordered, processed and reviewed followed similar trends, increasing in volume during late mornings and early afternoons, and tapering off during late-afternoon (Fig. 2b-d). This likely reflects the window of time whereby the bulk of routine clinical decision-making took place. We further observed that the average CRT and LTAT for individual tests varied by the time of day the tests were ordered, peaking at around 2 pm, coinciding with the time with the highest volume of tests ordered (Fig. 3). The high load of laboratory tests may have caused the delay in turnaround time, and as a result, laboratory tests that were ordered after 2 pm had a substantially longer delay in CRT and LTAT. These findings strongly suggest that clinical workflow and available resources played an important role in influencing when test results were processed and reviewed. Improving workflow and resource allocation may therefore offer an opportunity to reduce unnecessary hospital stay. For example, enhancing capacity planning to address variability in staffing workload, such as increasing resources during peak periods [16], and reviewing test results when they are available and discharging patients when they are clinically ready for discharge, regardless of the time of day, may improve patient flow and reduce unnecessary LOS. There are also potential opportunities for leveraging health informatics technology to facilitate the timely notification of available test results to providers more quickly. Any such interventions would need to ensure that providers are not unduly burdened with alarm fatigue and additional workload, and that the interventions do not lead to other unintended consequences.

### Study limitations

Our study is limited to a single center. Other centers may vary greatly in resources and organizational structure, which may in turn influence the relationship between delay in reviewing test results and LOS. Nonetheless, we believe that the organization of care in this teaching hospital is similar to other hospitals in most developed countries. To address the potential bias caused by variability in clinical workflow, we performed subgroup analyses of departments that served vastly different patient subpopulations within the hospital, including geriatrics, general surgery, cardiology and psychiatry. Additionally, subgroup analyses that considered patients with different principal diagnosis were conducted. The relationship between delay in test results follow-up and LOS was consistently observed in these analyses. Indeed, cumulative CRT and the number of tests performed were the only variables that were significantly associated with LOS across all settings and patient populations.

Our study cannot establish causality. While we have included potential confounders in our models, including disease severity and patient’s age, we could not account for other important factors such as staffing level, team rotations, and the behavior of individual providers – these factors can lead to delay in test results follow-up and directly impact the quality of care that results in prolonged hospitalization independent of delay in test results follow-up. Providers may also be less inclined to rapidly review test results if they expect patients to remain hospitalized due to their conditions. However, our analysis of the follow-up behavior of test results by day of admission suggests that on average, the overall test results follow-up patterns were not driven by the expected LOS. Furthermore, in sensitivity analyses that considered separately tests ordered within the first 2 days of admission and tests ordered after the first 2 days of admission, the association between delay in test results follow-up and LOS remained significant.

There is also limitation in the use of Charlson comorbidity index to adjust for disease severity, an important determinant of LOS. While Charlson comorbidity index – a weighted score of comorbidities based on their relative risk of mortality – has been extensively validated in published literature as a reliable prognostic indicator for mortality [17–20], its predictive power may vary across different patient subpopulations. To address this limitation, we conducted additional subgroup analyses for patients who were likely to have similar disease severity based on their principal reason for admission, including patients with renal failure and those who were admitted for an elective hip or knee replacement. In all analyses, the relationship between test follow-up delay and prolonged LOS was consistently observed. While further research is needed to understand the relationship between delay in reviewing test results and LOS and to account for unmeasured confounders, our unique data source that captures fine-grained timestamps of when tests were ordered, processed and reviewed, offer the rare opportunity to quantify delay in test results follow-up and to study its impact on LOS.

## Conclusions

Reducing unnecessary hospital LOS has become a critical health policy goal as the costs of healthcare escalate, and budgetary constraints in health care limit available resources. Our study highlights an important, yet underexplored, risk factor of prolonged LOS that warrants further investigation. Preventing delay in reviewing test results may provide a low-hanging fruit for ameliorating unnecessary hospital stays.

## Additional file

Additional file 1: **Figure S1.** Average clinical read time (CRT) for tests ordered on a given day of admission. (DOCX 100 kb)

## Abbreviations

CRT: Clinician read time
LOS: Length of stay
LTAT: Laboratory turnaround time
