# Supplementary figures and images for: Delay in reviewing test results prolongs hospital length of stay: a retrospective cohort study
Source: BMC Health Serv Res. 2018 May 16;18:369. doi: 10.1186/s12913-018-3181-z (PMC5956538; doi:10.1186/s12913-018-3181-z)

**Figure S1.** Average clinical read time (CRT) for tests ordered on a given day of admission


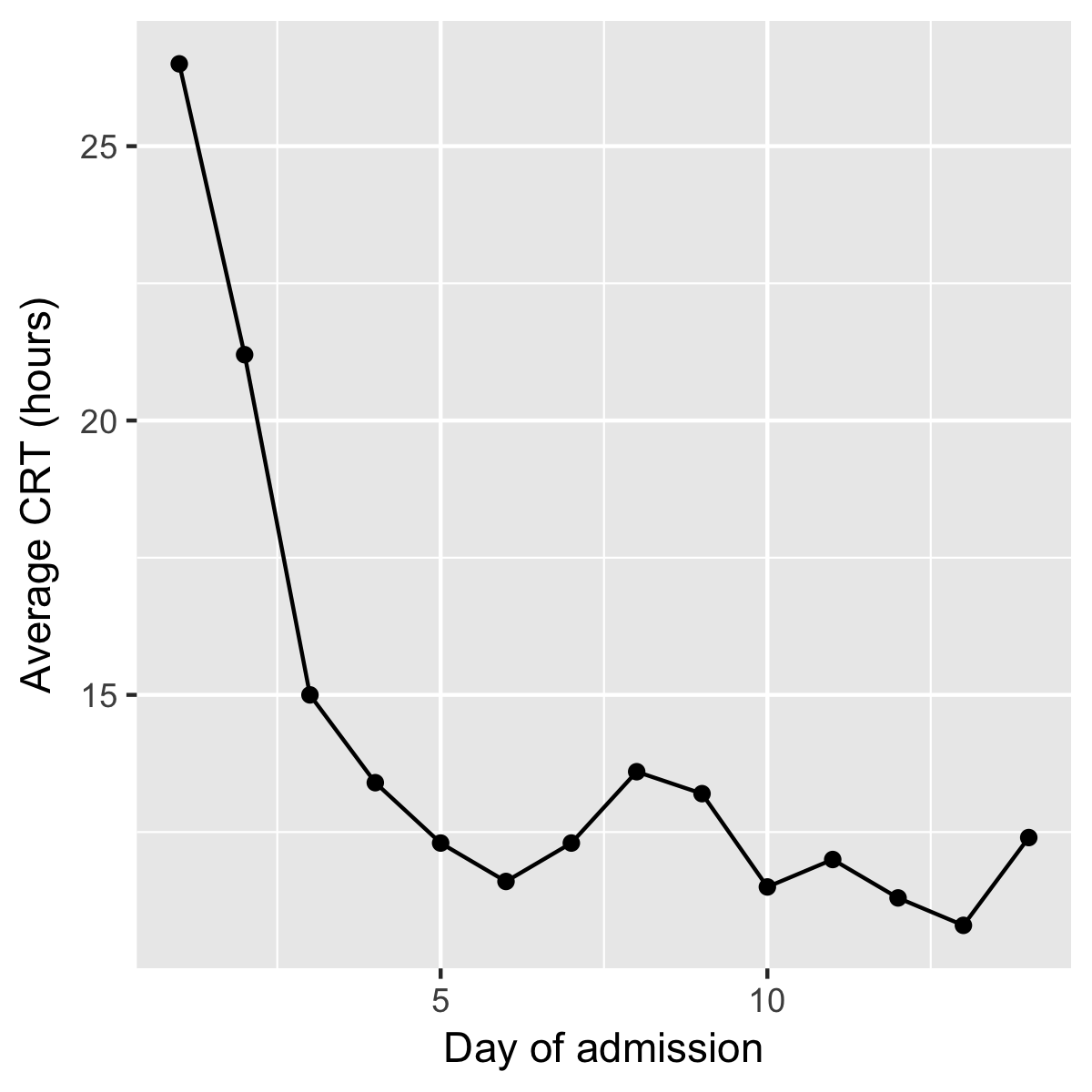

Supplement: Supplementary file 1 — Figure S1. Average clinical read time (CRT) for tests ordered on a given day of admission. (DOCX 100 kb) [file 12913_2018_3181_MOESM1_ESM.docx]
